# Supplementary material for: A New RNA-Dependent Cas12g Nuclease
Source: Int J Mol Sci. 2023 Dec 4;24(23):17105. doi: 10.3390/ijms242317105 (PMC10707612; doi:10.3390/ijms242317105)
Supplement: Supplementary file 1 [file Figure S1.pdf › Figure S1.pdf]

```
                     cov     pid   1 [        .         .         .         .         :         .         .         . 80
1 known           100.0% 100.0%    MAQASSTPAVSPRPRPRYREERTLVRKLLPRPGQSKQEFRENVKKLRKAFLQFNADVSGVCQWAIQFRPRYGKPAEPTET
2 our              98.7%  65.5%    MAQASLAAESQPFRRPRYREERTLVRRLLRFPGQSKEEFRQKIRQLREAFEQFNADVAGVCQWAIRFRPQSGQPAQPANE

                     cov     pid  81          .         1         .         .         .         .         :         . 160
1 known           100.0% 100.0%    FWKFFLEPETSLPPNDSRSPEFRRLQAFEAAAGINGAAALDDPAFTNELRDSILAVASRPKTKEAQRLFSRLKDYQPAHR
2 our              98.7%  65.5%    FWRFFLDPEAFVPTNDTRTPDFRRLQAFDAATGIGNLDELPDPPFTPALRASIESARNVPKTQEAQKLIARLKNYLLPHR

                     cov     pid 161          .         .         .         2         .         .         .         . 240
1 known           100.0% 100.0%    MILAKVAAEWIESRYRRAHQNWERNYEEWKKEKQEWEQNHPELTPEIREAFNQIFQQLEVKEKRVRICPAARLLQNKDNC
2 our              98.7%  65.5%    MIVLKAAAEWIQARYERGYQNWLRQYEEWEKEKQEWERTHPELTLEIRAAFNEIFQELKVKEKRPRICSLEHLQQNRDNC

                     cov     pid 241          :         .         .         .         .         3         .         . 320
1 known           100.0% 100.0%    QYAG------KNKHSVLCNQFNEFKKNHLQGKAIKFFYKDAEKYLRCGLQSLKPNVQGPFREDWNKYLRYMNLKEETLRG
2 our              98.7%  65.5%    QYAGEQPKRSKHRHSPLCLKFAEFCRQKLDKNAKKHFYKDAQQFLNKGLRSLKPRVQKSFKGNWHRYLQFMNLKENTIRK

                     cov     pid 321          .         .         :         .         .         .         .         4 400
1 known           100.0% 100.0%    KNGGRLPHCKNLGQECEFNPHTALCKQYQQQLSSRPDLVQHDELYRKWRREYWREPRKPVFRYPSVKRHSIAKIFGENYF
2 our              98.7%  65.5%    QYGGQLPHCEKLAQECRFNPHTELCKQYKSRLAGKPELARQEKLYREWRSNYWRGPRKPVFRYPSARLRSTPKIFGENYF

                     cov     pid 401          .         .         .         .         :         .         .         . 480
1 known           100.0% 100.0%    QADFKNSVVGLRLDSMPAGQYLEFAFAPWPRNYRPQPGETEISSVHLHFVGTRPRIGFRFRVPHKRSRFDCTQEELDELR
2 our              98.7%  65.5%    TADFQKSVVGLRLDSMPEGQYLEFAFAPWPRNYNPQPDQTTISSVHLHFAGTRPRIGFRFSVKHKASRFTCSQEELDELR

                     cov     pid 481          .         5         .         .         .         .         :         . 560
1 known           100.0% 100.0%    SRTFPRKAQDQKFLEAARKRLLETFPGNAEQELRLLAVDLGTDSARAAFFIGKTFQQAFPLKIVKIEKLYEQWPN-QKQA
2 our              98.7%  65.5%    SRRFPRQAQDQQFLDAARSRLLESFQGQPEQELRILAVDLGTSSAQAAFFVGKNCKQTHPLKIVKIEKLYEQPPKTDDTS

                     cov     pid 561          .         .         .         6         .         .         .         . 640
1 known           100.0% 100.0%    GDRRDASSKQPRPGLSRDHVGRHLQKMRAQASEIAQKRQELTGTPAPETTTDQAAKKATLQPFDLRGLTVHTARMIRDWA
2 our              98.7%  65.5%    GAAGDAGKKKYRLGLSKDHVGRHLETLGKKASAIAQKRNQLRNTSATAAQ--------TLKPFDLRGLAGHTARMIRDWA

                     cov     pid 641          :         .         .         .         .         7         .         . 720
1 known           100.0% 100.0%    RLNARQIIQLAEENQVDLIVLESLRGFRPPGYENLDQEKKRRVAFFAHGRIRRKVTEKAVERGMRVVTVPYLASSKVCAE
2 our              98.7%  65.5%    RLNARQIIALAEEKQADLIVLESLRGFRPPGYDNPDLEKKRRMAFFAYGRIRRKLAEKAVERGMRVVTVPYLYSSKVCSK

                     cov     pid 721          .         .         :         .         .     ] 776
1 known           100.0% 100.0%    CRKKQKDNKQWEKNKKRGLFKCE--GCGSQAQVDENAARVLGRVFWGEIELPTAIP
2 our              98.7%  65.5%    CGRRQDDEKRWNNNKLKGCFECEHRDCGYKAQADENAARVLGRVFWGEINLPAS--
```

Figure S1. *Cas12g sequence alignment. Aligned amino acid sequences of Cas12g proteins: known Cas12g and our Cas12g variant. The dash (-) sign indicates the absence of a homologous amino acid at this position in the second sequence. The cov value presents the percent of coverage of each sequence and pid value is the percent of identical amino acids. Sequence alignment was done with Mview web service at EMBL-EBI site.*
